# Health Behavior Changes Among Adults in the Supplemental Nutrition Assistance Program Education, Los Angeles County, California

**DOI:** 10.5888/pcd18.210221

**Published:** 2021-12-16

**Authors:** Julia I. Caldwell, Tony Kuo, Dipa Shah-Patel, Deborah A. Cohen

**Affiliations:** 1 Los Angeles County Department of Public Health, Los Angeles, California; 2 University of California, Los Angeles, California; 3 University of California, Clinical and Translational Science Institute, Los Angeles, California; 4 Kaiser Permanente Research and Evaluation, Pasadena, California

## Abstract

**Purpose and Objectives:**

The Supplemental Nutrition Assistance Program Education (SNAP-Ed), the educational branch of SNAP, can play an important role in improving dietary outcomes, eliminating food insecurity, and preventing chronic disease among low-income populations. This study examined the effects of local SNAP-Ed efforts on self-reported health behaviors and body mass index (BMI) over a 1-year period, using data collected from intercept surveys of program-eligible adults.

**Intervention Approach:**

From 2016 to 2020, the Los Angeles County Department of Public Health partnered with 24 community-based organizations to provide nutrition education and to implement policy, systems, and environmental changes in the community.

**Evaluation Methods:**

A cross-sectional survey was conducted in 2018 and repeated in 2019 to measure 6 outcomes describing population-level changes in health behaviors and BMI. The study recruited 4 samples: 2 samples from outside selected supermarkets (2018, n = 2,098; 2019, n = 2,323) and 2 samples from participants at SNAP-Ed class sites (2018, n = 651; 2019, n = 569).

**Results:**

While study results showed an increase in consumption of fruits and vegetables and in vigorous physical activity, they also showed an increase in BMI and high consumption of unhealthy foods. Participating in SNAP-Ed classes was positively associated with several health behaviors but no change in BMI. Participants who experienced food insecurity had worse health behavior outcomes than those who did not experience this condition.

**Implications for Public Health:**

SNAP-Ed interventions appear to have a favorable effect on fruit and vegetable consumption, but increases in BMI suggest that unhealthy food consumption is abundant and may be counteracting the benefits gained from eating more fruits and vegetables. Future efforts should take these results into consideration and optimize enrollment in nutrition assistance programs. These efforts should include coordinating with local programs to increase healthy food access for at-risk low-income populations in Los Angeles County.

**Summary**

**What is already known on this topic?**

The Supplemental Nutrition Assistance Program Education (SNAP-Ed) can improve dietary and physical activity outcomes. However, despite implementation of SNAP-Ed policy, systems, and environmental changes, the health effects of these interventions are not well understood.

**What is added by this report?**

Using intercept survey data collected at 2 time points, results showed positive effects of SNAP-Ed programming on several health behaviors but it fell short of eliminating the persistent effect of food insecurity.

**What are the implications for public health practice?**

In addition to increasing access to fresh produce and enrolling eligible populations in nutrition assistance programs, SNAP-Ed implementing agencies should work with partners to address social conditions, such as poverty.

## Introduction

Low-income populations are at greater risk for chronic disease because they face a disproportionately higher burden of food insecurity and prevalence of poor dietary consumption (1,2). The US Department of Agriculture (USDA) Supplemental Nutrition Assistance Program Education (SNAP-Ed), the educational branch of SNAP, is a $431 million program that implements interventions to improve diet and food security among low-income households that are eligible for SNAP. SNAP-Ed has more than 140 implementing agencies and hundreds of subcontractors throughout the US (3). Adults who are eligible for the program are those with annual household incomes less than or equal to 185% of federal poverty guidelines (4).

With funding from USDA and state-specific guidance from the California Department of Social Services and the California Department of Public Health, the Los Angeles County Department of Public Health (DPH) operates one of the largest SNAP-Ed programs in the nation. From 2016 to 2020, DPH partnered with 24 implementing agencies to advance policy, systems, and environmental change strategies (PSEs) and to provide nutrition education and promote physical activity in low-income communities in Los Angeles County (LAC). The implementation and evaluation of these efforts are focused on individual-level, direct-interaction nutrition education and physical activity promotion within the community.

Participation in a SNAP-Ed nutrition education class has been positively associated with participant nutrition-related self-efficacy, attitudes, and behaviors, such as incorporating fruits and vegetables into meal planning (4,5). Some studies indicate that nutrition education is associated with increased fruit and vegetable (FV) consumption, which is a priority outcome for SNAP and for SNAP-Ed (5,6). Participation in SNAP-Ed nutrition education classes has been shown to improve food security status (7,8).

A unique element of SNAP-Ed has been its goal to layer complementary PSEs alongside nutrition education (3). Examples of SNAP-Ed PSEs have included edible gardens in schools, incentive voucher programs in communities and health care settings, and healthy retail initiatives to promote FV consumption. Although states and implementing agencies have increasingly implemented PSEs throughout the past decade, limited research has characterized potential effects of SNAP-Ed programming at the community level (9). For example, 2 recent studies showed that for low-income caregivers of children who lived in high SNAP-Ed reach census tracts (versus low SNAP-Ed reach census tracts), PSEs were associated with increased FV consumption and decreased intake of sugar-sweetened beverages (SSBs) (10,11). In California, a population-level study demonstrated that SNAP-Ed–eligible populations increased their FV consumption over a 3-year period after exposure to PSEs (12). Other program evaluations show that socioeconomic status and place-based factors can influence dietary behaviors and health conditions. For instance, findings from a 2020 analysis of the California Health Interview Survey data suggest that SNAP-Ed–eligible adults from a low-income neighborhood consumed more SSBs in the past month and had a higher obesity risk than similar SNAP-Ed eligible adults from a high-income neighborhood (13). More research and evaluation is needed to build on this evidence and work toward a better understanding of how SNAP-Ed PSEs affect dietary behaviors and obesity risk from a population health perspective or at the program level. Data collected across multiple time points in the same population(s) would aid in this effort (14).

Our study sought to address gaps in SNAP-Ed programming by examining data from 2 waves of a cross-sectional intercept survey administered to SNAP-Ed–eligible adults in LAC during 2018 and then again in 2019. The resulting analysis describes SNAP-Ed PSEs and their potential influence on dietary behaviors and body mass index (BMI) for this urban sample.

## Purpose and Objectives

The 2016 to 2020 LAC SNAP-Ed program selected strategies to address diet-related chronic disease risk factors, such as healthy eating and physical activity, at 3 levels of the social-ecological model (15): individual, institutional, and environmental (Figure).

**Figure:**
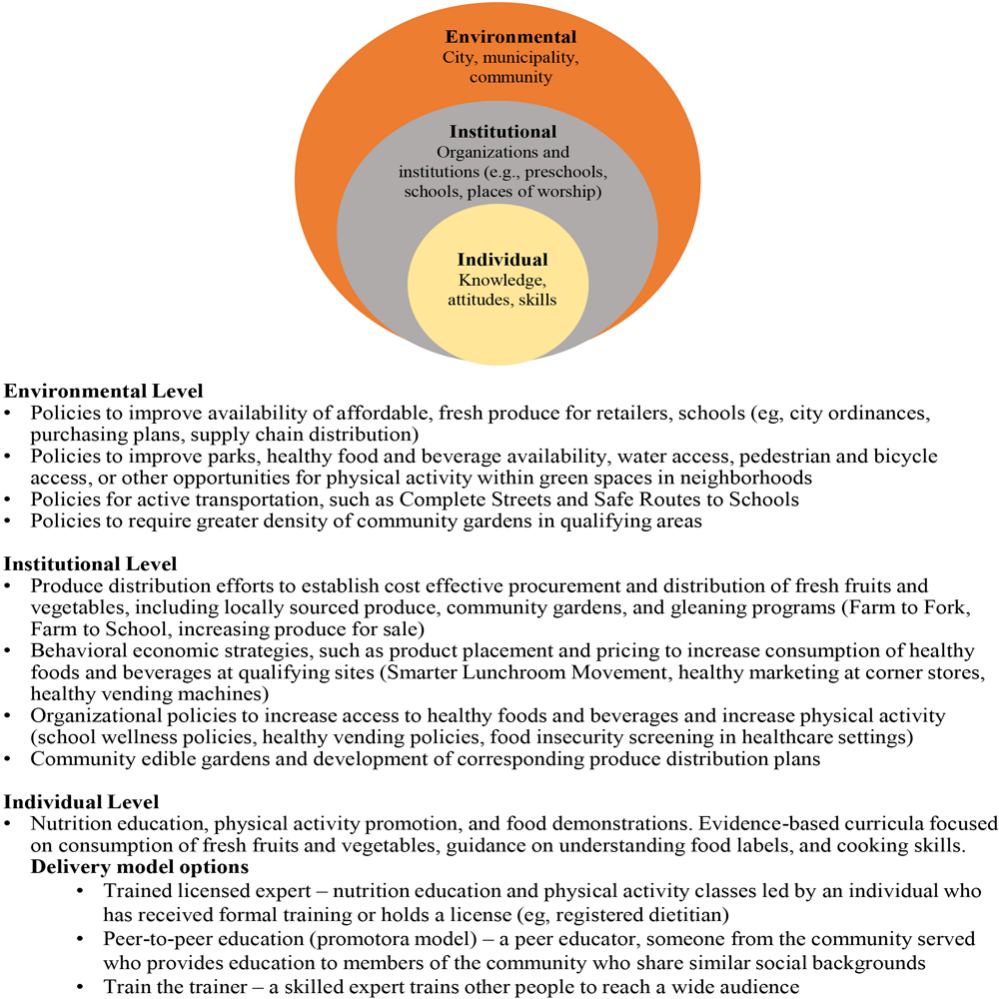
Social-ecological model implemented by the Supplemental Nutrition Assistance Program Education, Los Angeles County, 2016–2020.

This local program was developed using the USDA SNAP-Ed Plan Guidance, the USDA Evaluation Framework, and the Dietary Guidelines for Americans (15–17). Dietary Guidelines for Americans serves as the basis for the design of federal nutrition education materials and for nutrition education planning among USDA and US Health and Human Services nutrition programs. Additionally, state-specific guidance from the California Department of Social Services and the California Department of Public Health contributed to the scope of the LAC SNAP-Ed. The California Health Interview Survey, 2013–2015 datasets (18), helped identify the areas of greatest need for LAC populations, allowing DPH to make thoughtful resource allocation decisions about SNAP-Ed services. Throughout the process, DPH also relied on its previous experiences with SNAP-Ed funding to craft and update the local program so that it would deliver high quality nutrition education and PSEs across the region (19).

An important aspect of the local program was its commitment to partner with community-based agencies that work to improve food quality and food access in LAC. To accomplish this, DPH developed a request for proposals to select 24 organizations that were considered well positioned to reach low-income households throughout under-resourced communities in the county. These agencies were contracted to work within 1 of 8 service planning areas in the county based on their expertise, proposal quality, and population reach.

Overall, the evaluation of LAC SNAP-Ed PSEs and programming focused on demonstrating positive changes to USDA priority indicators, including FV consumption, physical activity level, and BMI (16). We designed the study as a brief intercept survey to collect information about these indicators and about the factors that might have influenced the dietary behaviors and BMI of SNAP-Ed–eligible adults. The survey was conducted in 2 waves. The first wave was administered in 2018 and the second wave in 2019. DPH contracted with Rand Corporation, an external evaluator, to carry out this task.

## Intervention Approach

From 2016 to 2020, the 24 implementing agencies delivered SNAP-Ed nutrition education and implemented PSEs in LAC. These agencies included 17 nonprofit organizations, 2 academic institutions, 3 health care systems, 1 faith-based organization, and 1 school district. All were tasked to carry out 1 required implementation action at each of the 3 levels of the social-ecological model: 1) individual-level nutrition education and physical activity promotion, 2) institutional PSEs, and 3) environmental PSEs. For levels 2 and 3, implementing agencies could choose from a menu of strategies selected by DPH. PSEs specific to LAC were selected and implemented across all 24 partners (Figure). This project was reviewed and approved by the Rand Corporation and the DPH institutional review boards.

Over the course of LAC SNAP-Ed, approximately 20,000 direct and indirect nutrition education and physical activity classes and promotional activities were delivered, reaching an estimated 2 million people. Because the SNAP-Ed reporting system did not fully delineate between first-time and repeat attendees, the reach number may contain duplicate program participant entries. To advance the PSEs, most of the funded partners either established new coalitions or joined existing coalitions to coordinate their work in the community. Edible gardens, healthy retail initiatives, and free produce distribution were the most frequently implemented institutional and environmental PSEs. Approximately 300 PSE-related projects were implemented or initiated at some level, reaching approximately 1.2 million people.

## Evaluation Methods

Our objective was to assess the effects of local SNAP-Ed programming, as indicated by changes in FV servings, SSB consumption, water consumption, energy dense–low nutrient food consumption, days of vigorous physical activity, and BMI. A questionnaire was developed and used to assess these individual-level changes and to estimate the population reach of the overall SNAP-Ed interventions. For the purposes of our study, improvements were defined as changes in the outcomes between 2018 and 2019, time points that account for the varied and broad range of PSEs that were implemented by the 24 implementing agencies.

### Data sources

The brief intercept survey collected 2 different adult samples at the 2 time points for a collective total of 4 samples. These samples comprised a general population of adults who were eligible for SNAP-Ed and a group of participants recruited from nutrition education classes that were provided by LAC’s implementing agencies (SNAP-Ed class sites). The intercept method of data collection has been successfully implemented in other studies to recruit low-income and diverse population samples (20,21).

The general population sample was selected among shoppers at 20 food retail outlets in 2018 and at 15 of the same outlets in 2019. Two large supermarket chains gave permission to contact their customers on the premises. Supermarkets were restricted to neighborhoods where at least 50% of the population in the store census tract was SNAP-Ed–eligible, defined by a household income of less than or equal to 185% of the federal poverty guidelines. SNAP-Ed nutrition education and programs are required to be delivered in these high-need census tracts. All shoppers going in and out of the selected supermarkets were approached and invited to complete a brief questionnaire. To be eligible for the study, participants had to reside in Los Angeles County, be 18 years or older, and speak English or Spanish. Participants or their child(ren) had to be enrolled in 1 of the following programs: SNAP, Special Supplemental Nutrition Program for Women, Infants, and Children (WIC), California Food Assistance Program (CFAP), California Work Opportunity and Responsibility to Kids (CalWORKs), General Relief, Child and Adult Care Food Program (CACFP), Head Start, Medicaid (California’s Medi-Cal program), Reduced Lunch, Section 8 housing, Summer Food Program, or Supplemental Security Income (SSI).

For the SNAP-Ed class site-specific sample, all program participants present on the day of data collection were asked to participate. Seventeen sites were included in the 2018 sample and 14 of the same sites were included in 2019. The study enrolled adults aged 18 years or older, so that only implementing agencies whose target population was adults participated in the study. Service planning area 5 (West LA) was excluded, as this region did not have any retail food outlets in neighborhoods where at least 50% of residents were SNAP-Ed-eligible.

The same questionnaire was administered at all sites in either English or Spanish. The questionnaire was brief, self-administered, and could be completed in 5 to 10 minutes. Questions assessed various health behaviors focusing on those most relevant to the SNAP-Ed mission as well as the interactions between study participants and the implementing agencies. All study participants were offered a $5 gift card.

The serial intercept survey used the same questionnaire administered in 2019 as 2018. Data collection occurred during March and April in both years. The only exceptions were that fewer supermarkets and fewer SNAP-Ed class sites were included in 2019, and all study interviewers or data collectors were bilingual in 2019. Five previously participating supermarkets did not renew permission to conduct these surveys, and 3 previously participating SNAP-Ed class sites no longer served adults by 2019 and were not eligible to participate in the second wave of the survey.

### Measures

Six dependent variables were selected, based on the SNAP-Ed Evaluation Framework (16). The first outcome variable, FV consumption, was created by combining 2 questions that asked participants, “In the last 24 hours, about how many servings of fruit did you eat?” and “In the last 24 hours, about how many servings of vegetables did you eat? Do not include potatoes.” One serving was defined for participants as “about the size of your fist.” For both questions, participants could select from 6 answers: none, less than 1, 1, 2, 3, or 4 or more servings. The second outcome, SSB consumption, asked participants, “On an average day, about how many sodas or sweetened drinks such as Gatorade, Red Bull or Sunny Delight do you drink? Do not include diet sodas or sugar-free drinks. Please count a 12-ounce can, bottle or glass as one drink.” Answer choices were none, less than 1, 1, 2, 3, 4, or 5 or more. The third outcome, servings of energy dense–low nutrient food, was created by combining 3 separate questions on reported servings of candy (about 1 medium Snickers bar per serving), cookies, and chips (1 handful is 1 serving) in the last 24 hours. Answer choices for all 3 questions were none, less than 1, 1, 2, 3, or 4 or more. The fourth outcome, water consumption, asked participants, “On average, how many cups of water (1 cup equals 8 ounces) do you usually drink in 1 day?” Answer choices were number of cups from 0 to 10. The fifth outcome was an adapted question (22) on vigorous physical activity and captured the days per week that the participant “did exercise or activities that required hard physical effort and caused heavy sweating and large increases in breathing and heart rate for at least 10 minutes without stopping.” Answer categories were none, 1, 2, 3, 4 or 5, or 6 or more days. The sixth outcome, BMI, was calculated from self-reported weight and height (calculated as weight in kilograms divided by the square of height in meters).

Participation in a SNAP-Ed class was captured by asking participants, “Not including today, in the past year, have you taken a nutrition, cooking, or physical activity class sponsored by any of the following Champions for Change groups?” with yes or no response options. Champions for Change was the name for SNAP-Ed in California at the time. For this question, the answer choices were specific to the geographic region where the survey was being administered and listed the name of each implementing agency who delivered services in that service planning area. This was done to help with name recognition and to reduce participant burden by not listing all 24 agencies.

The study covariates included age (continuous), sex (male or female), and race and ethnicity (African American, Asian or Pacific Islander, Latino or Hispanic, Non-Hispanic White, and other race, which included American Indian or Alaska Native, some other race or ethnicity, and multiracial). Educational attainment categories were less than high school, high school only, and more than high school. Other study covariates were the number of children in the household younger than age 18, whether the participant was a SNAP or CalFresh recipient, Medicaid or Medi-Cal recipient, or WIC recipient, and whether or not they completed the questionnaire in Spanish. Food insecurity was captured by using the 2-question food insecurity tool (23).

### Study analyses

Analyses were conducted by using the 2 samples obtained from the supermarkets and the 2 samples obtained from the SNAP-Ed class sites, and study investigators compared responses obtained in 2018 and 2019. Multivariable ordinary least squares regression models were constructed to predict the changes in consumption of FVs, SSBs, energy dense–low nutrient foods, and water; vigorous physical activity (level); and BMI. Outliers with BMI greater than 60 were excluded. The regression analyses controlled for demographic characteristics, public program participation (SNAP, Medicaid, WIC), Spanish language, and food insecurity status. Regression analyses also accounted for the clustering of responses by the supermarket and SNAP-Ed class site samples. All analyses were performed by using Stata version 14 (StataCorp, LLC).

## Results

In the 2018 supermarket sample, 2,098 of the 2,874 (73.0%) shoppers who were approached agreed to participate in the study and completed the intercept questionnaire. In the 2019 supermarket sample, 2,323 of the 3,037 (76.5%) shoppers who were approached agreed to participate and completed the intercept survey. In the 2018 SNAP-Ed class site sample, all 651 program participants (100.0%) who were approached agreed to participate and completed the intercept survey. In the 2019 SNAP-Ed class site sample, 569 of the 634 program participants (89.7%) who were approached agreed to participate and completed the intercept survey.

Compared with the 2018 supermarket sample, the 2019 supermarket sample was slightly older. The proportion of women was higher (70.3% vs 54.3%), as was the proportion of participants who were Latino or Hispanic, and the proportion of African Americans was smaller (Table 1). No significant changes in household food insecurity status were observed; 34.8% and 35.7% were food insecure in 2018 and 2019, respectively. A greater percentage of participants completed the survey in Spanish in 2019 than in 2018 (68.6% vs 46.3%). Across the 2 years, 13.8% of survey participants from the supermarket samples said they had heard of Champions for Change, the local program name of SNAP-Ed. Across the 2 years, 14.0% of survey participants from the supermarket samples said they had taken a nutrition, cooking, or physical activity class sponsored by a SNAP-Ed implementing agency in their local area.

**Table 1:** Demographic Characteristics of Survey Participants in Supermarket and SNAP-Ed Class Site Samples, Los Angeles County, California, 2018 and 2019

Characteristic	Supermarket Sample			SNAP-Ed Class Site Sample		
	2018	2019	*P* Value^a^	2018	2019	*P* Value^a^
**Study population**	2,098	2,323	NA	651	569	NA
**Number of data collection sites**	20	15	NA	17	14	NA
**Age, mean (SD)**	42.4 (15.1)	43.8 (14.0)	.002	44.5 (15.0)	46.3 (15.0)	.04
**Sex, %**						
Male	45.6	29.7	.001	18.1	16.4	.44
Female	54.3	70.3		81.9	83.6	
**Race or ethnicity, %**						
African American	27.0	15.1	.001	4.3	4.9	.38
Asian or Pacific Islander	1.1	0.7		2.3	3.5	
Non-Hispanic White	8.8	2.9		6.9	5.6	
Other^b^	9.8	4.0		4.6	3.2	
Latino or Hispanic	53.3	77.4		81.9	82.8	
**Educational attainment, %**						
Less than high school	29.3	40.3	.001	42.1	41.3	.03
High school only	39.2	34.9		23.1	29.4	
More than high school	31.5	24.8		34.9	29.4	
**Number of children, mean (SD)**	1.2 (1.6)	1.8 (1.7)	.001	1.7 (1.4)	1.8 (1.6)	.41
**SNAP recipient, %**	45.4	37.9	.001	34.4	35.5	.71
**Medicaid recipient, %**	60.2	65.7	.001	66.5	65.1	.63
**WIC recipient, %**	13.5	15.1	.14	19.2	20.8	.50
**Spanish language questionnaire, %**	46.3	68.6	<.001	69.4	70.8	.61
**Food insecure^c^, %**	34.8	35.7	.50	57.8	43.8	<.001
**Took at least 1 class^d^, %**	15.4	12.6	.007	47.9	30.3	<.001

Abbreviations: NA, not applicable; SNAP-Ed, Supplemental Nutrition Assistance Program Education; WIC, Special Supplemental Nutrition Program for Women, Infants, and Children.

a Chi-square tests (categorical variables) and *t* tests (continuous variables) were used to compare 2018 to 2019.

b Includes American Indian or Alaska Native, some other race or ethnicity, and multiracial.

c In the past 12 months, either often or sometimes: 1. I worried about whether food would run out before having money to buy more, or 2. The food did not last and they did not have money to get more.

d “Not including today, in the past year, have you taken a nutrition, cooking, or physical activity class sponsored by any of the following Champions for Change groups?” The names of each agency who delivered services in that service planning area were then listed.

Survey participants from the SNAP-Ed class site samples were similar in 2018 and in 2019; however, they were older and a higher proportion completed high school in 2019. A smaller proportion of participants were food insecure in 2019 than in 2018 (43.8% vs 57.8%). Across the 2 years, 40.0% of participants had previously taken at least 1 program class.

In the supermarket samples, FV consumption increased from an average of 3.3 cups in 2018 to 3.6 cups in 2019 (*P* = .001) (Table 2). While the average number of servings of SSBs and energy dense–low nutrient foods decreased between the years, on average, participants continued to report consuming at least 1 soda and more than 4 servings of candy, cookies, or chips in the last 24 hours. Average number of days of vigorous physical activity in a week was higher in 2019 (2.0 d vs 1.8 d in 2018, *P* = .001). Average BMI increased between the years. Among the SNAP-Ed class site samples, a small increase in the average of SSB consumption was observed from 2018 to 2019. Average BMI also increased in these samples, as did average number of days of vigorous exercise (2.0 d vs 2.3 d, *P* = .002).

**Table 2:** Unadjusted Comparisons of Participant Health Behaviors and BMI Between Supermarket and SNAP-Ed Class Site Samples, Los Angeles County, California, 2018 and 2019

Behavior	Supermarket Sample			SNAP-Ed Class Site Sample		
	2018	2019	*P* Value^a^	2018	2019	*P* Value^a^
	Mean (SD)	Mean (SD)		Mean (SD)	Mean (SD)	
Servings of fruits and vegetables per day	3.3 (2.5)	3.6 (2.6)	.001	4.2 (2.5)	4.0 (2.5)	.17
Servings of sugar-sweetened beverages per day	1.5 (1.6)	1.3 (1.4)	.001	0.8 (1.0)	0.9 (1.2)	.03
Servings of water per day	5.3 (2.7)	5.3 (2.7)	.93	5.6 (2.7)	5.4 (2.6)	.24
Servings of energy dense–low nutrient foods per day^b^	4.6 (4.0)	4.1 (3.8)	.001	3.4 (2.9)	3.2 (2.8)	.30
Days of vigorous physical activity per week	1.8 (2.0)	2.0 (2.0)	.001	2.0 (1.9)	2.3 (2.0)	.002
BMI^c^	27.5 (5.4)	29.6 (6.5)	.001	27.6 (5.2)	29.2 (6.3)	.001

Abbreviations: BMI, body mass index; SNAP-Ed, Supplemental Nutrition Assistance Program Education.

a *t* tests were used to compare 2018 with 2019.

b In the last 24 hours, sum of reported candy servings (about 1 Snickers bar per serving), cookies, and chips (1 handful is 1 serving).

c Self-reported weight and height were used to calculate BMI (weight in kilograms divided by the square of height in meters).

The multivariable regression analyses showed that survey participants from the supermarket samples consumed 0.22 more servings of FV (*P* = .012) and reported 0.34 more days of vigorous physical activity (*P* < .001) from 2018 to 2019 (Table 3). Participants in the supermarket samples gained 1.76 BMI points over time (*P* < .001). Among those who participated in a SNAP-Ed class, the increase in FV consumption and vigorous physical activity was of a greater magnitude than that for the other behavior categories. Participating in a class was also associated with an increase in servings of water. Among those who participated in a class, no significant change in BMI was observed. Food insecurity was associated with lower FV consumption, more SSB consumption, more energy dense–low nutrient food consumption, and fewer days of vigorous physical activity.

**Table 3:** Changes in Health Behaviors and BMI^a ^Over Time in Supermarket Sample, Los Angeles County, California, 2018 and 2019

Characteristic	Servings of Fruit and Vegetables, Coef (*P* Value)	Servings of Sugar-Sweetened Beverages, Coef (*P* Value)	Servings of Water, Coef (*P* Value)	Servings of Energy Dense–Low Nutrient Foods^b^ (*P* Value)	Days of Vigorous Physical Activity (*P* Value)	BMI (*P* Value)
**Change over time**	0.22 (.012)	−0.05 (.32)	−0.01 (.89)	0.00 (.96)	0.34 (<.001)	1.76 (<.001)
**SNAP-Ed participation**						
≥1 class	0.33 (.006)	−0.05 (.47)	0.28 (.03)	0.23 (.20)	0.40 (<.001)	0.38 (.22)
No classes	1 [Reference]	1 [Reference]	1 [Reference]	1 [Reference]	1 [Reference]	1 [Reference]
**Age**	0.01 (.001)	−0.01 (<.001)	0.00 (.35)	−0.03 (<.001)	−0.01 (.010)	0.02 (.02)
**Sex**						
Female	0.39 (<.001)	−0.21 (<.001)	−0.06 (.54)	−0.79 (<.001)	−0.25 (.001)	0.80 (<.001)
Male	1 [Reference]	1 [Reference]	1 [Reference]	1 [Reference]	1 [Reference]	1 [Reference]
**Race or ethnicity**						
African American	0.17 (.14)	0.31 (<.001)	0.06 (.65)	0.97 (<.001)	0.15 (.10)	−0.23 (.43)
Asian or Pacific Islander	0.64 (.13)	−0.25 (.33)	0.72 (.13)	0.37 (.56)	0.58 (.10)	−2.07 (.055)
Non-Hispanic White	0.08 (.66)	0.82 (<.001)	−0.18 (.39)	1.20 (.001)	0.34 (.03)	−0.66 (.16)
Other^c^	0.07 (.67)	0.16 (.12)	−0.04 (.82)	0.47 (.07)	0.08 (.54)	0.09 (.83)
Latino or Hispanic	1 [Reference]	1 [Reference]	1 [Reference]	1 [Reference]	1 [Reference]	1 [Reference]
**Education**						
Less than high school	1 [Reference]	1 [Reference]	1 [Reference]	1 [Reference]	1 [Reference]	1 [Reference]
High school	0.06 (.57)	0.02 (.78)	0.10 (.38)	0.26 (.09)	0.14 (.10)	−0.08 (.75)
More than high school	0.40 (.001)	−0.18 (.01)	0.31 (.02)	−0.16 (.39)	0.53 (<.001)	0.32 (.30)
**No. of children**	0.06 (.014)	0.04 (.005)	0.04 (.14)	0.08 (.04)	0.01 (.67)	0.08 (.27)
**SNAP recipient**						
Yes	−0.10 (.23)	0.22 (<.001)	0.00 (.96)	0.35 (.006)	–0.03 (.62)	0.30 (.16)
No	1 [Reference]	1 [Reference]	1 [Reference]	1 [Reference]	1 [Reference]	1 [Reference]
**Medicaid recipient**						
Yes	−0.05 (.55)	−0.07 (.17)	0.22 (.02)	−0.34 (.01)	−0.04 (.59)	0.27 (.23)
No	1 [Reference]	1 [Reference]	1 [Reference]	1 [Reference]	1 [Reference]	1 [Reference]
**WIC recipient**						
Yes	0.27 (.03)	0.04 (.60)	0.03 (.84)	−0.05 (.77)	0.01 (.88)	−0.01 (.97)
No	1 [Reference]	1 [Reference]	1 [Reference]	1 [Reference]	1 [Reference]	1 [Reference]
**Spanish language questionnaire**						
Yes	−0.08 (.43)	−0.12 (.04)	−0.02 (.87)	−0.41 (.006)	−0.01 (.87)	0.27 (.28)
No	1 [Reference]	1 [Reference]	1 [Reference]	1 [Reference]	1 [Reference]	1 [Reference]
**Food insecure**						
Yes	−0.42 (<.001)	0.28 (<.001)	−0.21 (.07)	0.92 (<.001)	−0.16 (.050)	0.17 (.52)
No	1 [Reference]	1 [Reference]	1 [Reference]	1 [Reference]	1 [Reference]	1 [Reference]
**Sample size**	3,702	3,755	3,731	3,700	3,558	3,207
***R* ^2^**	0.03	0.06	0.01	0.06	0.03	0.04

Abbreviations: BMI, body mass index; coef, coefficient; SNAP-Ed, Supplemental Nutrition Assistance Program Education; WIC, Special Supplemental Nutrition Program for Women, Infants, and Children.

a Self-reported weight and height were used to calculate BMI (weight in kilograms divided by the square of height in meters).

b Sum of reported servings of candy (about 1 medium Snickers bar per serving), cookies, and chips (1 handful is 1 serving) in the last 24 h.

c Includes American Indian or Alaska Native, some other race or ethnicity, and multiracial.

 The multivariable regression analyses from the SNAP-Ed class site samples showed that survey participants reported 0.50 more days of vigorous physical activity (*P* = .001) yet consumed 0.15 more servings of SSBs (*P* = .046) from 2018 to 2019 (Table 4). Participants in the SNAP-Ed samples gained 1.30 BMI points over time (*P* = .001). Taking more than 1 SNAP-Ed class, however, was not associated with a significant change in BMI and was not associated with increases in energy dense–low nutrient consumption. Taking more than 1 class was associated with an increase in FV consumption and physical activity. Food insecurity was associated with lower FV consumption, greater SSB, and energy dense–low nutrient food consumption.

**Table 4:** Changes in Health Behaviors and BMI^a^ Over Time in the SNAP-Ed Class Site Sample, Los Angeles County, California, 2018 and 2019

Variables	Servings of Fruit and Vegetables, Coef (*P* Value)	Servings of Sugar-Sweetened Beverages, Coef (*P* Value)	Servings of Water, Coef (*P* Value)	Servings of Energy Dense–Low Nutrient Foods^b^, Coef (*P* Value)	Days of Vigorous Physical Activity, Coef (*P* Value)	BMI, Coef (*P* Value)
**Change over time**	−0.12 (.46)	0.15 (.046)	−0.04 (.81)	−0.10 (.61)	0.50 (.001)	1.30 (.001)
**SNAP-Ed participation**						
≥1 class	0.54 (.001)	−0.04 (.55)	0.29 (.10)	−0.09 (.64)	0.43 (.002)	−0.39 (.34)
No classes	1 [Reference]	1 [Reference]	1 [Reference]	1 [Reference]	1 [Reference]	1 [Reference]
**Age**	0.01 (.23)	−0.01 (<.001)	0.00 (.57)	−0.03 (<.001)	0.00 (.67)	0.01 (.41)
**Sex**						
Female	0.75 (<.001)	−0.25 (.004)	−0.07 (.75)	−0.23 (.34)	−0.17 (.33)	0.32 (.51)
Male	1 [Reference]	1 [Reference]	1 [Reference]	1 [Reference]	1 [Reference]	1 [Reference]
**Race and ethnicity**						
African American	−0.11 (.77)	0.61 (<.001)	0.59 (.16)	1.28 (.004)	−0.19 (.57)	0.61 (.53)
Asian or Pacific Islander	0.62 (.22)	0.07 (.72)	−0.13 (.80)	1.20 (.03)	0.94 (.02)	−3.28 (.004)
Non-Hispanic White	0.02 (.97)	0.10 (.52)	−0.44 (.26)	0.28 (.49)	−0.31 (.30)	−1.07 (.21)
Other^c^	0.16 (.70)	−0.14 (.40)	−0.59 (.17)	0.12 (.80)	0.53 (.11)	0.86 (.38)
Latino or Hispanic	1 [Reference]	1 [Reference]	1 [Reference]	1 [Reference]	1 [Reference]	1 [Reference]
**Educational attainment**						
Less than high school	1 [Reference]	1 [Reference]	1 [Reference]	1 [Reference]	1 [Reference]	1 [Reference]
High school only	0.27 (.19)	−0.08 (.37)	−0.06 (.77)	−0.15 (.50)	−0.08 (.63)	−0.39 (.44)
More than high school	0.18 (.40)	−0.23 (.009)	−0.01 (.98)	0.01 (.97)	−0.20 (.26)	−0.58 (.26)
**No. of children**	0.03 (.64)	0.03 (.23)	0.14 (.02)	-0.03 (.69)	0.03 (.52)	0.40 (.006)
**SNAP recipient**						
Yes	−0.06 (.74)	0.16 (.03)	0.16 (.36)	0.34 (.08)	0.04 (.79)	1.04 (.01)
No	1 [Reference]	1 [Reference]	1 [Reference]	1 [Reference]	1 [Reference]	1 [Reference]
**Medicaid recipient**						
Yes	0.00 (.98)	0.04 (.58)	0.14 (.43)	0.27 (.15)	−0.07 (.59)	0.06 (.89)
No	1 [Reference]	1 [Reference]	1 [Reference]	1 [Reference]	1 [Reference]	1 [Reference]
**WIC recipient**						
Yes	0.70 (.001)	−0.04 (.66)	0.10 (.63)	0.25 (.29)	−0.02 (.92)	−0.69 (.17)
No	1 [Reference]	1 [Reference]	1 [Reference]	1 [Reference]	1 [Reference]	1 [Reference]
**Spanish language questionnaire**						
Yes	−0.08 (.67)	−0.07 (.43)	−0.31 (.16)	0.10 (.67)	−0.04 (.81)	−1.33 (.007)
No	1 [Reference]	1 [Reference]	1 [Reference]	1 [Reference]	1 [Reference]	1 [Reference]
**Food insecure**						
Yes	−0.45 (.009)	0.41 (<.001)	−0.31 (.08)	1.16 (<.001)	−0.23 (.09)	0.06 (.88)
No	1 [Reference]	1 [Reference]	1 [Reference]	1 [Reference]	1 [Reference]	1 [Reference]
**Sample size**	1,014	1,025	1,022	1,017	936	899
***R* ^2^**	0.05	0.12	0.02	0.08	0.03	0.06

Abbreviations: BMI, body mass index; SNAP-Ed, Supplemental Nutrition Assistance Program Education; WIC, Special Supplemental Nutrition Program for Women, Infants, and Children.

a Self-reported weight and height were used to calculate BMI (weight in kilograms divided by the square of height in meters).

b Sum of reported servings of candy (about 1 medium Snickers bar per serving), cookies, and chips (1 handful is 1 serving) in the last 24 h.

c Includes American Indian or Alaska Native, some other race or ethnicity, and multiracial.

## Implications for Public Health

Our study describes the effects of local SNAP-Ed efforts on self-reported health behaviors, including physical activity and BMI, by using a population sample of adults eligible for the SNAP-Ed program as well as a sample of adults receiving nutrition education at SNAP-Ed class sites. The overall findings of benefits versus no change in behaviors or obesity risk were mixed. Improvements in health behaviors were reported in 2018 and 2019, including up to half a day more of vigorous physical activity in the past week, and a quarter serving more of FV consumption in the past day among the general population eligible for the SNAP-Ed program. Despite favorable findings, participants continued to consume at least 1 SSB a day and over 4 servings of energy dense–low nutrient foods a day across the 2 years. BMI increased over time for all study populations. Participating in a SNAP-Ed class was associated with higher FV consumption, water consumption, more vigorous physical activity, and no significant change in BMI. Adults who experienced food insecurity had worse health behavior outcomes, after controlling for public program participation and SNAP-Ed class participation.

The increase in FV consumption and vigorous physical activity over time could potentially be attributed to the implementation of PSEs at the local level. A varied and broad range of PSEs were implemented at that time by local agencies in the county, including edible gardens, healthy retail initiatives, and free produce distributions. Other research has shown an increase in FV consumption among SNAP-Ed eligible populations in California (12). These positive behavioral changes, however, might also be the result of other local and national obesity and chronic disease prevention programs, including the National Diabetes Prevention Program, which uses similar diet and physical activity interventions (24).

Despite observing positive changes in health behaviors, consumption of SSBs and energy dense–low nutrient foods remained high, and BMI increased in the study samples. In California, other work indicates that over a recent 3-year period, SSB consumption was unchanged for low-income mothers, adolescents, and children (11). Nationally, intake of SSBs remained high particularly for racial and ethnic minorities (25). A possible reason that BMI increased in our study is that local SNAP-Ed interventions may have focused primarily on consumption of healthy foods, with less attention on reducing SSBs or energy dense–low nutrient foods, which are known contributors to weight gain (26). In more recent efforts, SNAP-Ed in California has implemented the Rethink Your Drink educational campaign that encourages water consumption over SSB consumption. During the time of data collection, experiences of stress, particularly for those with limited resources, may have also increased. Stress has been shown to be associated with obesity (27). Experiences of stress may have increased for the recruited study populations, given that most were Latino and Spanish speaking. During the time of data collection, changes were proposed in the Public Charge rule that threatened to refuse citizenship to immigrants who took advantage of public welfare programs like SNAP and SNAP-Ed (28).

The reach of local implementing agencies could have been limited. At the time of data collection, the SNAP-Ed population in LAC was estimated to be more than 3.5 million in a county of more than 10 million people. LAC SNAP-Ed direct and indirect nutrition education and physical activity classes reported reaching 2 million people. This number, however, includes program participants who may have attended multiple times. Implementing agencies at the local level are small, and many methods of engagement resulted in smaller classes and PSE changes, such as community gardens, which are difficult to scale. The environmental conditions and strong marketing of unhealthy foods (29) might have also made it difficult for SNAP-Ed PSEs to truly influence change.

A promising finding from our study was the observed benefits associated with participating in SNAP-Ed classes, particularly for FV consumption, a SNAP-Ed priority indicator (16). Those who took at least 1 class had no significant change in BMI. A general trend of increasing participation in physical activity that was enhanced by taking a class was also reported. These findings support previous work that suggests SNAP-Ed nutrition education classes are positively associated with nutrition-related behaviors and FV consumption (4–6). In the supermarket sample, 14% said they had taken a class sponsored by a SNAP-Ed implementing agency in their local area. To increase favorable effects, local SNAP-Ed implementing agencies could consider coordinating with other local programs that work to increase access to produce for low-income populations including market match and produce prescription programs.

Food insecurity appeared to have a substantially negative influence on selected health behaviors, including consuming more SSBs and energy dense–low nutrient foods. Of all study participants, 35% to 58% reported some level of household food insecurity in the past 12 months. In 2018, an estimated 26.8% of LAC households with incomes less than 300% of the federal poverty guidelines were food insecure, according to a population-based telephone questionnaire (30). While SNAP-Ed classes can help improve food security status (7,8), stress may contribute to worsening health behaviors despite participant knowledge about its negative health effects (31). Programs and interventions should work to integrate and maximize enrollment of eligible populations into nutrition assistance programs including SNAP, WIC, and senior meal programs. More policy and programmatic work are needed to address the structural inequities that contribute to obesity and hunger. To do that, county and local agencies should play a more proactive role in coalition efforts that seek to address broader inequalities in poverty, housing, and food insecurity.

Our study has several limitations. Health behavior outcomes and BMIs may be subject to recall bias. Social desirability bias, particularly at the SNAP-Ed site locations, may have affected reporting of these health indicators. Our analyses were unable to capture the direct effects of PSEs on self-reported health behaviors because the questionnaire did not specifically ask participants directly about exposure to a PSE. Data on whether participants were pregnant or breastfeeding also were not collected, which may have played a role in the reporting of dietary consumption and calculation of BMI. A significant strength of the study is the in-person intercept survey design, which captured a hard-to-reach population. The study had a strong response rate, ranging from 73% to 100%, depending on the sample location and year of data collection. Future studies could benefit from following a cohort of program-eligible adults over time and documenting any interaction(s) with SNAP-Ed PSEs.

SNAP-Ed can play an important role in helping to improve dietary and physical activity outcomes, thereby facilitating opportunities to prevent chronic disease among hard-to-reach, low-income populations. While direct nutrition education is a primary component of SNAP-Ed, delivery of classes can be resource intensive and limited in its scope and reach. Given that millions of people in LAC are eligible for the SNAP-Ed program, scaling complementary PSEs to address structural conditions that can contribute to inequities will be a critical undertaking for the county of Los Angeles, as its health and social services agencies look for cost-effective and sustainable ways to improve health in the community. Future research and evaluation should build on this and other evidence for a better understanding of how SNAP-Ed PSEs affect dietary behaviors and obesity risk from a population health perspective or at the program level.
